# Dietary Sheep Blood Zymolysate Modulates Gut Barrier Integrity, Microbiota Composition, and Nutritional Metabolism in Common Carp (*Cyprinus carpio*)

**DOI:** 10.1155/anu/7862053

**Published:** 2026-09-11

**Authors:** Jingjing Bao, Yujun Jiang, Xusheng Hao, Xuelian Feng, Mengyuan Zhang, Yina Qiao, Xin Hou, Yilin Yang, Xiaoyi Xu, Yingchun Liu, Yuhong Zhang, Feng Gao

**Affiliations:** ^1^ College of Animal Science, Inner Mongolia Agricultural University, Hohhot, Inner Mongolia, China, imau.edu.cn; ^2^ College of Life Science, Inner Mongolia Agricultural University, Hohhot, Inner Mongolia, China, imau.edu.cn; ^3^ Institute of Biotechnology, Chinese Academy of Agricultural Sciences, Beijing, China, caas.cn

**Keywords:** common carp, growth performance, intestinal barrier function, microbiota modulation, sheep blood zymolysate

## Abstract

Sustainable protein sources are pivotal to the green development of aquaculture. As an underutilized abattoir byproduct, sheep blood holds great potential for valorization via enzymatic hydrolysis. This study evaluated the impacts of dietary sheep blood zymolysate (SBZ) supplementation (0.3%, 0.6%, and 1.0% dosages, denoted as TL, TM, and TH groups, respectively) on growth performance, serum biochemistry, intestinal barrier function, and gut microbiota of common carp (*Cyprinus carpio*). A total of 240 carp (initial weight: 48.0 ± 0.2 g) were randomly assigned to four groups (three replicates/group and 20 fish/replicate) with a basal diet as control (CON) for an 8‐week feeding trial. Results demonstrated that TL and TM groups exhibited significantly higher average daily gain (ADG), weight gain rate (WGR), and specific growth rate (SGR) than the CON group (*p*  < 0.01). The TM group (0.6% SBZ) showed the most prominent benefits: It had elevated whole‐body crude protein content (*p*  = 0.037), serum albumin (ALB) and total protein (TP) levels (*p*  < 0.0001 and *p* = 0.028, respectively), and intestinal villus height (VH) (*p*  < 0.0001), along with upregulated expression of intestinal tight junction genes (*ZO-1*, *Occludin*, and *Claudin-1*) and nutrient transporter genes (excitatory amino acid transporter [*EAAT*], cationic amino acid transporter [*CAT*], and peptide transporter 1 [*PepT1*]) (*p*  < 0.01). Additionally, TH group (1.0% SBZ) increased muscle histidine (His) content, while TM and TH groups enhanced muscle arginine (Arg) levels. At the microbial level, SBZ supplementation significantly enriched the relative abundance of phylum *Pseudomonadota* and genus *Gemmobacter* and reshaped the gut microbial community structure. Overall, dietary SBZ supplementation improves carp growth, body protein deposition, intestinal morphology, and barrier integrity and modulates gut microbiota, with 0.6% SBZ being the optimal dosage for integrated performance. This study provides empirical support for the high‐value utilization of sheep blood byproducts in sustainable aquafeed formulation.

## 1. Introduction

Blood from livestock slaughter is internationally recognized as a high‐quality protein source, containing 17%–22% crude protein rich in essential amino acids and bioactive peptides [1]. Despite its nutritional value, ovine blood—generated in large volumes worldwide—remains vastly underutilized, with less than 30% converted into feed‐grade blood meal. At the same time, the rest is discarded, leading to significant nutrient loss and environmental pollution [2]. Current blood processing often remains rudimentary, hindered by collection inefficiencies and underdeveloped technologies for maximizing functional properties. Enzymatic hydrolysis emerges as a key strategy to transform blood, particularly sheep blood—a major byproduct in regions like Inner Mongolia—into zymolysates with enhanced bioavailability and bioactivity, offering both ecological and economic benefits for the feed, food, and pharmaceutical sectors.

Common carp is a globally significant aquaculture species due to its hardiness, omnivorous diet, and economic value. Optimizing feed formulations for carp is crucial for sustainable aquaculture growth. Fishmeal is still indispensable in carp diets; however, supplementing functional additives beyond the basal formulation can further improve the performance. Blood enzymatic zymolysates possess bioactive peptides that may serve this role, yet their effects in common carp remain unexplored.

Protein zymolysates, produced via enzymatic digestion, offer a superior alternative. Hydrolysis breaks down proteins into smaller peptides and free amino acids, enhancing digestibility and absorption [3]. Crucially, these zymolysates often possess bioactive properties beyond basic nutrition, including antioxidant, antimicrobial, anti‐inflammatory, and immunomodulatory activities [4]. Zymolysates derived from various animal, plant, and microbial sources have demonstrated benefits in aquaculture species, improving growth performance [5], enhancing protein metabolism and oxygen transport capacity (reflected in blood parameters) [6], and promoting intestinal health [7, 8]. Intestinal health is paramount in fish, encompassing efficient nutrient absorption (linked to villus architecture), robust barrier function (mediated by tight junction proteins (TJPs) like *ZO-1*, *Occludin*, and *Claudins*) [9, 10], and a balanced gut microbiota [11]. Zymolysates can positively influence these aspects by modulating gene expression of nutrient transporters (e.g., peptide transporter 1 [*PepT1*] and excitatory amino acid transporter [*EAAT*]) [12] and TJPs [13] and shaping microbial communities. Despite the recognized potential of blood zymolysates and their application in terrestrial livestock [14], research on sheep blood zymolysate (SBZ) in aquatic species, particularly in omnivorous fish like common carp, remains critically underexplored. This represents a significant gap, given the abundance of sheep blood waste in key production regions and the need for sustainable aquafeeds.

However, despite its potential, the comprehensive effects of SBZ on the gut barrier integrity, microbial ecosystem, and systemic nutrient metabolism in omnivorous fish, particularly common carp, remain largely unknown. Therefore, this study aimed to comprehensively evaluate the effects of dietary SBZ supplementation on common carp. This research provides essential data for the high‐value utilization of sheep blood byproducts in aquaculture feeds, contributing to resource efficiency, environmental sustainability, and the development of functional feed ingredients for improved carp health and productivity.

## 2. Materials and Methods

### 2.1. Animal Ethics Statement

All the experimental procedures applied in this study were reviewed and approved by the Animal Care and Use Committee of Inner Mongolia Agricultural University (Approval Number NND 2024036).

### 2.2. Experimental Design, Animals, and Management

Two hundred and forty healthy common carp, weighing 48 ± 0.2 g and displaying uniform size and robust condition, were randomly allocated to four dietary treatments (*n* = 60 fish per treatment). Each treatment comprised three replicates of 20 fish stocked at a density of 20 g/L in a 60‐L glass aquaria. Culture water consisted of continuously aerated municipal tap water maintained at 20–24°C, pH 7.4–7.8, dissolved oxygen ≥ 5.0 mg/L, and total ammonia nitrogen < 0.3 mg/L. Dietary regimens: control [CON]: basal diet (CON), TL: basal diet + 0.3% SBZ, TM: basal diet + 0.6% SBZ, and TH: basal diet + 1.0% SBZ. All diets were manufactured as 3‐mm sinking pellets. Feed formulation and nutritional composition are shown in Table 1. Fish were hand‐fed to apparent satiation twice daily (09:00 and 16:00) at 3% of biomass per day. The trial comprised a 14‐day acclimation period, followed by a 56‐day feeding phase. Throughout, water temperature was monitored daily, and aquaria were siphoned and exchanged each morning. Feed intake was recorded daily. At the end of the feeding phase, 12 fish per treatment (four per replicate) were randomly selected for sampling.

**Table 1:** Experimental basic diet and nutritional level (%, based on dry matter).

Ingredients	Content
Fish meal	10.00
Barley	19.00
Soybean meal	30.00
Rapeseed meal	25.00
Cotton meal	5.50
Soybean oil	5.50
Bentonite	2.00
Premix^a^	1.00
Calcium dihydrogen phosphate	2.00
Total	100.00
Nutritional level	
Crude protein	34.02
Crude fat	8.67
Crude fiber	5.33
Calcium	1.08
Phosphorus	1.32
Lysine	1.80
Methionine	0.59

^a^Each kilogram of premix contains the following ingredients: vitamin A, 800,000 IU; vitamin D, 180,000 IU; vitamin E, 4000 mg; vitamin K3, 200 mg; vitamin B1, 300 mg; vitamin B2, 1000 mg; vitamin B3, 1800 mg; vitamin B6, 450 mg; vitamin B12, 1.5 mg; vitamin C, 4500 mg; pantothenic acid, 900 mg; folic acid, 150 mg; choline, 55,000 mg; anhydrous copper sulfate, 1.8 g; anhydrous ferrous sulfate, 22 g; anhydrous zinc sulfate, 20 g; anhydrous manganese sulfate, 6 g; sodium selenite, 0.035 g; potassium iodide, 0.024 g; and cobalt chloride, 0.09 g.

### 2.3. Sample Collection and Measurement Methods

#### 2.3.1. Growth and Slaughter Performance

At trial initiation, initial body weights were recorded. After a 56‐day feeding phase, fish were fasted for 24 h; on the sampling day, 12 individuals per treatment were euthanized and individually weighed for final body mass. Growth indices were computed as follows: average daily gain (ADG), weight gain rate (WGR), specific growth rate (SGR), and condition factor (CF). Posteuthanasia, the hepatopancreas, kidney, and spleen were excised and weighed to determine the hepatosomatic (HSI), nephrosomatic (NSI), and splenosomatic (SSI) indices. The entire intestine was then divided into anterior, mid‐, and posterior segments; segmental mass and length were recorded to calculate intestinal weight and length indices.

#### 2.3.2. Proximate Composition of Dorsal Muscle and Whole Body

From each treatment, 12 fish were randomly selected; the left dorsal muscle was excised, pooled, and lyophilized. Essential and nonessential amino acids were quantified by automated ion‐exchange chromatography (Amino Acid Analyzer, Hitachi L‐8900, Japan) following acid hydrolysis (6 M HCl, 110°C, 22 h).

Nine fish per treatment were euthanized and stored at –40°C. After freeze‐drying (LGJ‐200F, Songyuan, China), samples were pulverized and analyzed in triplicate according to AOAC (1995): moisture: vacuum freeze‐drying to constant weight, crude protein Kjeldahl nitrogen × 6.25 (KjelFlex K‐360, Büchi, Switzerland), crude lipid Soxhlet extraction with petroleum ether (SOX‐406, Hanon, China), and ash charred on a hot plate, then ashed at 550°C to constant weight (4 h).

#### 2.3.3. Routine Blood and Serum Biochemical

Following a 24‐h fast, 12 carp per treatment were anesthetized, and blood was drawn from the caudal vein. After 30 min of clotting at ambient temperature, serum was separated by centrifugation (3500 rpm and 10 min) and stored at –20°C. Serum aspartate aminotransferase (AST), alanine aminotransferase (ALT), albumin (ALB), total cholesterol (CHOL), triglycerides (TGs), total protein (TP), lactate dehydrogenase (LDH), creatinine (CRE), glucose (GLU), high‐density lipoprotein CHOL (HDL‐C), and low‐density lipoprotein CHOL (LDL‐C) were quantified by an automated clinical chemistry analyzer (Hitachi 3110, Japan) using commercially available kits (Lepu Biotech, Beijing).

#### 2.3.4. Histological Examination of the Posterior Intestine

Posterior intestinal segments fixed in 10% neutral‐buffered formalin were trimmed, dehydrated, cleared, and embedded in paraffin. Serial sections (5 µm) were stained with hematoxylin and eosin (H&E) and mounted on glass slides. Digital images were acquired at 4× and 10× magnification using a whole‐slide scanner (Grundium, Finland). For each slide, three nonoverlapping fields were randomly selected. Villus height (VH) and crypt depth (CD) were measured with ImageJ (NIH, USA), and the VH‐to‐CD (V/C) ratio was calculated.

#### 2.3.5. Quantitative RT‐Polymerase Chain Reaction (PCR) Analysis of Tight‐Junction and Peptide‐Transporter Genes

Total RNA was extracted from the posterior intestinal tissue using the AccuratePure Tissue RNA Kit (Accurate Biology, China). First‐strand cDNA was synthesized with AccurateScript Reverse Transcriptase (Accurate Biology) and 1 µg RNA. Real‐time quantitative PCR was performed in duplicate with SYBR Premix Dimer Eraser (Sangon Biotech, Shanghai) on a CFX96 system (Bio‐Rad, USA). Gene‐specific primers (Table 2) were designed against NCBI sequences and synthesized by Sangon Biotech; β‐actin served as the endogenous control. Relative expression was calculated by the 2^–ΔΔCt^ method.

**Table 2:** Primer sequences of target genes.

Target gene	Forward primer sequences (5′ to 3′)	Reverse primer sequences (5′ to 3′)
β‐Actin	GATTGGCTGTGAGATGATGCT	CGTTGTAGAAGGTGTGATGCC
ZO‐1	TCTCCAGCCACTCTCCACAACAG	GATACGCTCCTCCTCACGACTCC
Occludin	GACCGCTAATGGGAGGAACC	GAGCTAAAGACACTGCCCGT
Claudin‐1	GAGTGCTCCATGTGAGAGGTG	CTGAAGAGCCATGGAGTTGGA
EAAT	CACAGCAACAGTCGCCAGCAT	TTCCAGCACCGTAGGCATCTCC
CAT	CCCGACATCACTGAACCCTCCA	GCACAGGCTGAACAGGACACTG
PepT1	GGCAACATCCTGCTGCAAGTCA	TTCTCTTCCGCCCAGTCCATCC

#### 2.3.6. Identification of Intestinal Microbial Communities

Microbial genomic DNA extraction from all samples was performed using the E.Z.N.A. Soil DNA Kit (Omega Bio‐tek, Norcross, USA), strictly adhering to the supplier’s protocol. The integrity of the extracted DNA was verified through electrophoresis on a 1% agarose gel. A NanoDrop 2000 spectrophotometer (Thermo Scientific, USA) assessed DNA concentration and purity. The full‐length bacterial 16S rRNA gene underwent amplification using purified DNA as the template. Universal primer pairs 338F (5′‐ACTCCTACGGGGAGGCAGCAG‐3′) and 806R (5′‐GGACTACHVGGGGTWTCTAAT‐3′) facilitated this amplification.

Each PCR, with a 20‐µL volume, contained the following components: 4 µL of 5× FastPfu buffer, 2 µL of dNTPs (2.5 mM concentration), 0.8 µL of each primer (5 µM), 0.4 µL of FastPfu polymerase, 0.2 µL of BSA, and template DNA (10 ng). Three separate PCR reactions were conducted for every sample. Amplification cycling, executed on a Bio‐Rad T100 Thermal Cycler (USA), proceeded under these conditions: initial denaturation at 95°C for 3 min; followed by 27 cycles of denaturation (95°C, 30 s), primer annealing (60°C, 30 s), and extension (72°C, 30 s); concluding with a final extension step at 72°C for 10 min; and a holding temperature of 4°C.

The resulting PCR amplicons underwent separation via 2% agarose gel electrophoresis. Purification was subsequently achieved using magnetic beads. Quantification of purified amplicons employed fluorometry on a Qubit 4.0 system (Thermo Fisher, USA). Samples were combined in equimolar ratios before sequencing.

Bioinformatic analysis leveraged the UPARSE 7.1 pipeline. This involved clustering sequences into operational taxonomic units (OTUs) at 97% similarity and the removal of chimeric sequences. To mitigate the influence of uneven sequencing depth on α‐ and β‐diversity assessments, data were subsampled (rarefied) to 6000 sequences per specimen, yielding a mean Good’s coverage estimate of 99.09%. Taxonomy classification was assigned using the RDP Classifier (v2.11) referencing the Silva 16S rRNA database (v138), applying a confidence threshold of 70%. Computational procedures were carried out on the Meggie BioCloud platform (https://cloud.majorbio.com).

### 2.4. Statistical Analysis

All data were analyzed with SAS 9.2 (SAS Institute, USA). Results are presented as the mean and standard error of the mean (SEM). All data were first tested for normality using the Shapiro–Wilk test and for homogeneity of variances via the Levene test. After confirming compliance with the assumptions of normality and homoscedasticity, one‐way analysis of variance (one‐way ANOVA) followed by Tukey’s multiple comparison test was performed for statistical comparison; *p*  < 0.05 was deemed significant. In tables, means within a column sharing the same superscript letter are not significantly different (*p*  > 0.05), adjacent letters denote significant differences (*p*  < 0.05), and nonadjacent letters indicate highly significant differences (*p*  < 0.01). Histograms were generated with GraphPad Prism 9.5; asterisks denote  ^∗^ *p*  < 0.05 and  ^∗∗^ *p*  < 0.01.

## 3. Results

### 3.1. Growth Performance

The impact of dietary SBZ supplementation on the growth performance of common carp is presented in Table 3. The results demonstrate that the inclusion of SBZ in the diet significantly enhanced growth metrics. Specifically, carp in the TL and TM groups exhibited markedly higher (*p*  < 0.01) FBW, ADG, WGR, and SGR when compared to the CON group. In contrast, no significant differences (*p*  > 0.05) in these parameters were observed between the TH group and the CON group.

**Table 3:** Effect of sheep blood zymolysate on growth performance of carp.

Item	CON	TL	TM	TH	SEM	*p*‐Value
IBW, g	48.17	48.15	48.28	48.27	0.13	0.829
FBW, g	57.30^a^	64.48^c^	66.75^c^	62.96^ac^	1.26	0.004
ADG, g/day	0.16^a^	0.29^c^	0.33^c^	0.26^ac^	0.02	0.006
WGR, %	18.95^a^	33.92^c^	38.27^c^	30.43^ac^	2.79	0.007
SGR, %/day	0.31^a^	0.52^c^	0.58^c^	0.47^ac^	0.04	0.006
CF, g/cm^3^	1.34	1.47	1.47	1.42	0.05	0.322

*Note:* CON, basal diet (control); TL, basal diet + 0.3% SBZ; TM, basal diet + 0.6% SBZ; TH, basal diet + 1.0% SBZ. Within a row, means with no common superscript letters differ significantly (*p* < 0.05).

### 3.2. Slaughter Performance

The effect of dietary SBZ supplementation on visceral organ indices in common carp is detailed in Table 4. The results indicate that SBZ inclusion in the diet did not significantly affect (*p*  > 0.05) the hepatopancreatic index or spleen index. However, compared to the CON group, the renal index was significantly increased (*p*  < 0.01) in the TL, TM, and TH groups.

**Table 4:** Effect of sheep blood zymolysate on organ index of carp.

Item	CON	TL	TM	TH	SEM	*p*‐Value
Hepatopancreatic index, %	1.93	1.82	2.00	2.00	0.12	0.673
Spleen index, %	0.31	0.40	0.44	0.39	0.04	0.230
Kidney index, %	0.31^a^	0.48^c^	0.48^c^	0.46^c^	0.02	<0.001

*Note:* CON, basal diet (control); TL, basal diet + 0.3% SBZ; TM, basal diet + 0.6% SBZ; TH, basal diet + 1.0% SBZ. Within a row, means with no common superscript letters differ significantly (*p* < 0.05).

The influence of SBZ on intestinal somatic indices and intestinal length indices is presented in Table 5. Results demonstrate that SBZ supplementation had no significant effect (*p*  > 0.05) on the somatic index of the posterior intestine or the length indices of the anterior, middle, or posterior intestine. Notably, the somatic index of the anterior intestine was significantly increased (*p*  < 0.05) in the TM and TH groups compared to the CON group. Although the TL group exhibited a tendency towards an increased anterior intestinal somatic index, this difference was not statistically significant (*p*  > 0.05).

**Table 5:** Effect of sheep blood zymolysate on intestinal index of carp.

Item	CON	TL	TM	TH	SEM	*p*‐Value
Foregut body index, %	0.80^a^	0.87^ab^	0.95^b^	0.95^b^	0.04	0.046
Midgut body index, %	0.36^a^	0.44^ab^	0.47^b^	0.43^ab^	0.03	0.013
Hindgut body index, %	1.17	1.21	1.30	1.27	0.09	0.687
Foregut length index, %	22.65	22.59	23.40	20.97	1.06	0.434
Midgut length index, %	18.31	18.38	18.39	18.27	0.95	0.996
Hindgut length index, %	72.68	73.32	75.96	72.78	3.98	0.929

*Note:* CON, basal diet (control); TL, basal diet + 0.3% SBZ; TM, basal diet + 0.6% SBZ; TH, basal diet + 1.0% SBZ. Within a row, means with no common superscript letters differ significantly (*p* < 0.05).

### 3.3. Proximate Composition of Dorsal Muscle and Whole Body

Table 6 summarizes the effects of dietary SBZ on essential amino acids in the dorsal muscle. Relative to CON, only the TH diet elevated His (*p*  < 0.05); TL and TM were unchanged. No SBZ level altered threonine, cysteine, valine, methionine, isoleucine, leucine, tyrosine, phenylalanine, or lysine (*p*  > 0.05).

**Table 6:** Effect of sheep blood zymolysate on essential amino acids in carp muscle.

Item	CON	TL	TM	TH	SEM	*p*‐Value
Thr, g/100 g	0.66	0.69	0.69	0.67	0.03	0.840
Cys, g/100 g	0.23	0.23	0.23	0.24	0.01	0.740
Val, g/100 g	0.69	0.72	0.71	0.69	0.03	0.805
Met, g/100 g	0.37	0.37	0.39	0.42	0.02	0.291
Ile, g/100 g	0.62	0.68	0.64	0.63	0.03	0.422
Leu, g/100 g	1.15	1.20	1.17	1.18	0.04	0.895
Tyr, g/100 g	0.45	0.47	0.46	0.48	0.02	0.900
Phe, g/100 g	0.64	0.68	0.66	0.65	0.02	0.759
Lys, g/100 g	1.38	1.44	1.45	1.41	0.06	0.864
His, g/100 g	0.44^a^	0.48^ab^	0.46^a^	0.53^b^	0.02	0.015

*Note:* CON, basal diet (control); TL, basal diet + 0.3% SBZ; TM, basal diet + 0.6% SBZ; TH, basal diet + 1.0% SBZ. Within a row, means with no common superscript letters differ significantly (*p* < 0.05).

Table 7 shows the corresponding data for nonessential amino acids. Dietary SBZ did not affect aspartate, serine, glutamate, glycine, or alanine (*p*  > 0.05). Arginine (Arg) was, however, increased by both TM and TH diets (*p*  < 0.05), whereas TL remained similar to CON (*p*  > 0.05).

**Table 7:** Effect of sheep blood zymolysate on nonessential amino acids in carp muscle.

Item	CON	TL	TM	TH	SEM	*p*‐Value
Asp, g/100 g	1.45	1.51	1.50	1.48	0.05	0.893
Ser, g/100 g	0.58	0.60	0.61	0.59	0.02	0.785
Glu, g/100 g	2.11	2.20	2.19	2.15	0.08	0.869
Gly, g/100 g	0.62	0.64	0.65	0.62	0.02	0.837
Ala, g/100 g	0.86	0.89	0.91	0.86	0.03	0.688
Arg, g/100 g	1.07^a^	1.12^ab^	1.17^b^	1.17^b^	0.03	0.032

*Note:* CON, basal diet (control); TL, basal diet + 0.3% SBZ; TM, basal diet + 0.6% SBZ; TH, basal diet + 1.0% SBZ. Within a row, means with no common superscript letters differ significantly (*p* < 0.05).

Table 8 presents the whole‐body proximate composition. Moisture, crude lipid, and ash were unaltered by SBZ supplementation (*p*  > 0.05). Crude protein was significantly elevated only in the TM group (*p*  < 0.05); TL and TH did not differ from CON (*p*  > 0.05).

**Table 8:** Effect of sheep blood zymolysate on nutrient composition of whole carp.

Item	CON	TL	TM	TH	SEM	*p*‐Value
Moisture, %	39.53	40.60	38.59	38.12	2.25	0.868
Crude protein, %	60.43^a^	60.48^a^	65.56^b^	62.92^ab^	1.45	0.037
Crude lipid, %	8.63	7.83	10.12	9.38	1.69	0.853
Ash, %	14.60	12.03	13.80	13.14	1.18	0.503

*Note:* CON, basal diet (control); TL, basal diet + 0.3% SBZ; TM, basal diet + 0.6% SBZ; TH, basal diet + 1.0% SBZ. Within a row, means with no common superscript letters differ significantly (*p* < 0.05).

Table 9 summarizes the textural profile of the carp muscle. Hardness was numerically greater in TM and TH than in CON and TL, but the difference did not attain significance (*p*  > 0.05). Springiness was uniformly maintained between 0.80 and 0.84 across treatments (*p*  > 0.05). Cohesiveness in TM exceeded that in all other groups (*p*  = 0.042), whereas gumminess was significantly higher in TM compared with TL (*p*  = 0.044). Chewiness was likewise elevated in TM relative to both CON and TL (*p*  = 0.046), and resilience was markedly superior in TM versus TL and TH (*p*  = 0.007).

**Table 9:** Effect of sheep blood zymolysate on the texture characteristics of carp muscle.

Item	CON	TL	TM	TH	SEM	*p*‐Value
Hardness/g	726.55	701.71	931.05	844.25	80.38	0.168
Springiness/mm	0.81	0.80	0.84	0.82	0.02	0.330
Cohesiveness	0.62^ab^	0.61^b^	0.65^a^	0.60^b^	0.01	0.042
Gumminess/g	451.76^ab^	422.19^b^	602.90^a^	512.52^ab^	51.48	0.044
Chewiness/mJ	369.33^b^	333.66^b^	502.62^a^	421.98^ab^	43.15	0.046
Resilience	0.24^ab^	0.23^bc^	0.27^a^	0.22^bc^	0.01	0.007

*Note:* CON, basal diet (control); TL, basal diet + 0.3% SBZ; TM, basal diet + 0.6% SBZ; TH, basal diet + 1.0% SBZ. Within a row, means with no common superscript letters differ significantly (*p* < 0.05).

### 3.4. Serum Biochemical

Table 10 details the effect of dietary SBZ supplementation on serum biochemical indices in common carp. Supplementation had no significant impact (*p*  > 0.05) on AST, ALT, total CHOL, TGs, LDH, CHOL ester (CE), GLU, high‐density lipoprotein (HDL), or low‐density lipoprotein (LDL). ALB levels were significantly elevated (*p*  < 0.01) in the TM and TH groups compared to CON, while the TL group showed no significant difference (*p*  > 0.05). TP levels were significantly elevated (*p*  < 0.05) in the TM group, while no significant differences (*p*  > 0.05) were observed in the TL and TH groups compared to CON.

**Table 10:** Effect of sheep blood zymolysate on serum biochemical indexes of carp.

Item	CON	TL	TM	TH	SEM	*p*‐Value
AST, U/L	82.49	95.27	92.76	85.05	8.44	0.670
ALT, U/L	2.94	3.25	3.18	3.33	0.22	0.630
ALB, g/L	10.02^a^	11.23^ab^	13.08^cd^	13.48^d^	0.52	<0.001
CHOL, mmol/L	2.47	2.36	2.62	2.61	0.12	0.409
TG, mmol/L	1.96	1.79	2.11	2.08	0.10	0.141
TP, g/L	26.55^a^	27.18^ab^	31.74^b^	31.06^ab^	1.62	0.028
LDH, U/L	246.94	222.17	209.92	211.50	26.90	0.751
CRE, µmol/L	43.86	43.95	41.50	40.33	3.54	0.856
GLU, mmol/L	4.26	4.11	4.87	4.53	0.59	0.806
HDL, mmol/L	2.65	2.86	3.00	2.83	0.24	0.781
LDL, mmol/L	2.28	2.37	2.80	2.86	0.20	0.094

*Note:* CON, basal diet (control); TL, basal diet + 0.3% SBZ; TM, basal diet + 0.6% SBZ; TH, basal diet + 1.0% SBZ. Within a row, means with no common superscript letters differ significantly (*p* < 0.05).

### 3.5. Histological Examination of the Posterior Intestine

**Figure 1:**
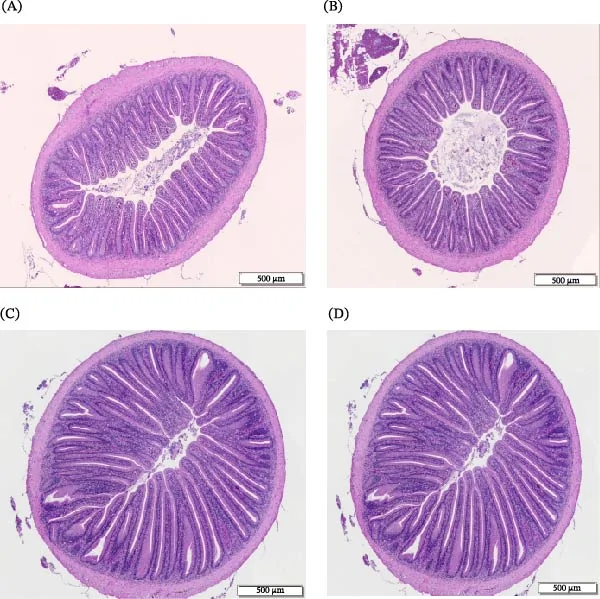
Carp intestinal tissue section (4×): (A) CON, (B) TL, (C) TM, and (D) TH.

**Figure 2:**
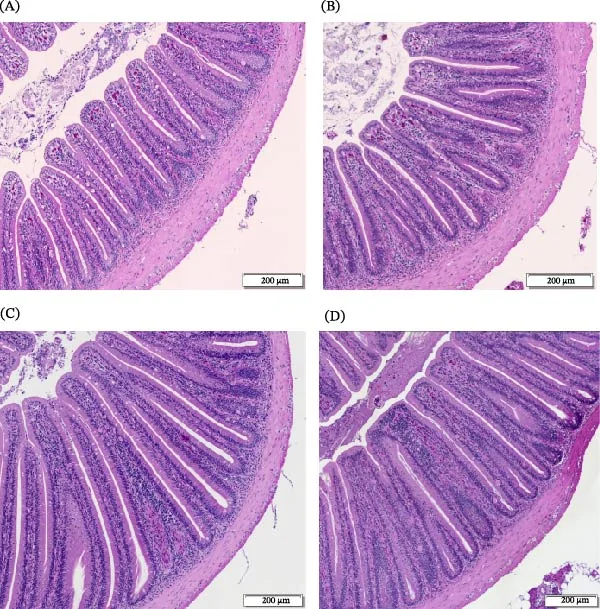
Carp intestinal tissue sections (10×): (A) CON, (B) TL, (C) TM, and (D) TH.

Table 11 presents the effect of dietary SBZ supplementation on intestinal morphology in common carp. VH was not significantly altered (*p*  > 0.05) in the TL group but was highly significantly elevated (*p*  < 0.01) in the TM and TH groups compared to CON. CD was highly significantly reduced (*p*  < 0.01) in the TL and TM groups and significantly reduced (*p*  < 0.05) in the TH group. The V/C ratio was highly significantly elevated (*p*  < 0.01) in all SBZ‐supplemented groups (TL, TM, and TH). The impact of SBZ on the intestinal structure is further illustrated in Figures 1 and 2. Compared to the CON group, intestinal villi appeared elongated and crypts shallower in all SBZ treatment groups. Villus architecture demonstrated enhanced structural integrity and more tightly arranged organization in SBZ‐fed carp.

**Table 11:** Effect of sheep blood zymolysate on the intestinal structure of carp.

Item	CON	TL	TM	TH	SEM	*p*‐Value
Villus height, µm	308.89^a^	324.85^a^	411.34^c^	390.91^c^	9.83	<0.001
Crypt depth, µm	68.77^a^	45.26^c^	38.96^c^	55.43^bc^	4.29	<0.001
V/C, %	6.37^a^	11.67^c^	15.95^e^	12.08^c^	1.00	<0.001

*Note:* CON, basal diet (control); TL, basal diet + 0.3% SBZ; TM, basal diet + 0.6% SBZ; TH, basal diet + 1.0% SBZ. Within a row, means with no common superscript letters differ significantly (*p* < 0.05).

### 3.6. Quantitative RT‐PCR Analysis of Tight‐Junction and Peptide‐Transporter Genes

**Figure 3:**
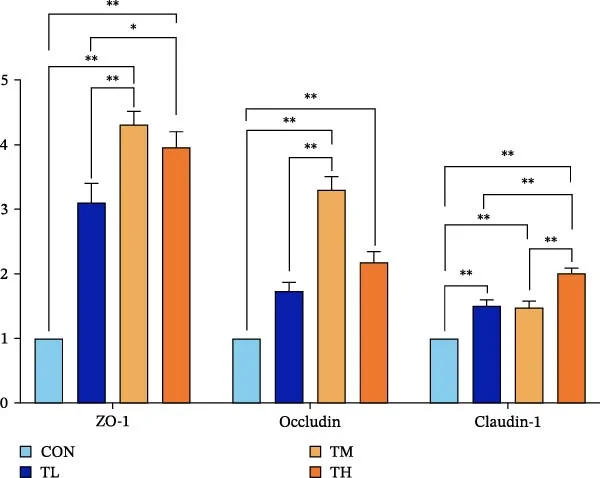
Effect of sheep blood zymolysate on intestinal tight junction protein gene expression in carp.  ^∗^ *p* < 0.05 and  ^∗∗^ *p* < 0.01.

Figure 3 illustrates the effects of dietary SBZ on intestinal TJP gene expression in common carp. Compared with the CON group, supplementation significantly increased *ZO-1* gene expression in the TM and TH groups (*p*  < 0.01), while no significant effect was observed in the TL group (*p*  > 0.05); significant differences were noted between TL and TM groups (*p*  < 0.01) and between TL and TH groups (*p*  < 0.05). *Occludin* expression significantly increased in TM and TH groups (*p*  < 0.01) with no change in TL (*p*  > 0.05), while TL and TM groups differed significantly (*p*  < 0.01). *Claudin-1* expression increased significantly in all supplemented groups (TL, TM, and TH; *p*  < 0.01), with significant differences between TL and TH (*p*  < 0.01) and between TM and TH (*p*  < 0.01).

**Figure 4:**
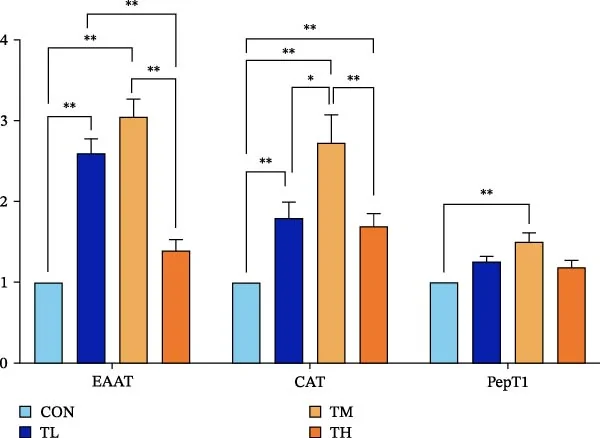
Effect of sheep blood zymolysate on gene expression of intestinal protein transporter vectors in carp.  ^∗^ *p* < 0.05 and  ^∗∗^ *p* < 0.01.

Figure 4 demonstrates the effects on peptide‐transporter gene expression. *EAAT* expression significantly increased in TL and TM groups (*p*  < 0.01) compared to CON, while TH showed no significant change (*p*  > 0.05); intergroup comparisons revealed significant differences between TL and TM (*p*  < 0.01), TL and TH (*p*  < 0.01), and TM and TH (*p*  < 0.01), with TL and TH also exhibiting significant differences (*p*  < 0.05). Cationic amino acid transporter [*CAT*] expression significantly increased in all supplemented groups (TL, TM, and TH; *p*  < 0.01), with significant differences between TL and TM (*p*  < 0.05) and between TM and TH (*p*  < 0.01). *PepT1* expression significantly increased only in the TM group (*p*  < 0.01) compared to CON, with no significant difference between TL and TH groups (*p*  > 0.05).

### 3.7. Identification of Intestinal Microbial Communities

**Figure 5:**
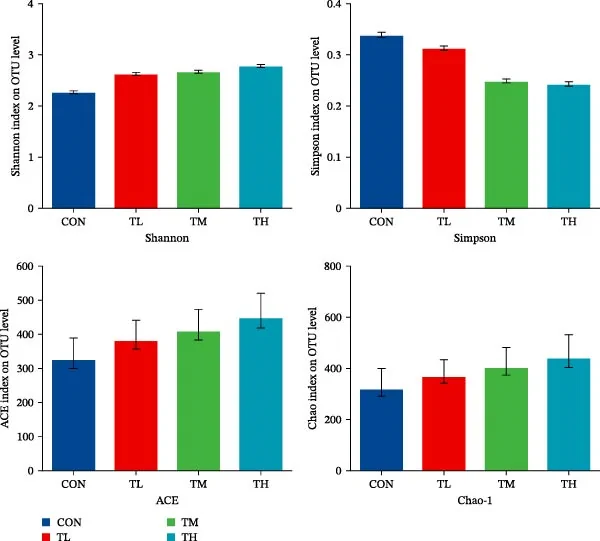
Effect of sheep blood zymolysate on intestinal α‐diversity in carp.

Alpha‐diversity metrics are provided in Figure 5. ACE, Chao1, and Shannon indices trended upwards in SBZ groups, while Simpson values declined, yet none of these shifts reached significance (*p* > 0.05). Figure 6A shows the impact of SBZ on the intestinal microbiome. Across all four groups, 478 OTUs were shared, whereas 92, 105, 281, and 140 OTUs were unique to CON, TL, TM, and TH, respectively. SBZ‐supplemented diets significantly elevated OTU richness relative to CON, with pronounced intertreatment divergence, indicating a pronounced modulatory effect on gut microbial communities that may carry physiological or pathophysiological relevance.

Figure 6(A) Effect of sheep blood zymolysate on intestinal microbial composition of carp. (B) Effect of sheep blood zymolysate on intestinal β‐diversity in carp. (C) Relative abundance of intestinal microbiota phylum level in carp. (D) Bacterial species with varying abundances at the phylum level of intestinal microbial communities in carp. (E) Relative abundance of intestinal microbiota genus level in carp. (F) Bacterial species with varying abundances at the genus level of intestinal microbial communities in carp. (G) Correlation analysis between intestinal microbiota and intestinal indexes in carp (genus level). (H) Correlation analysis of intestinal microbiota and muscle amino acids in carp (genus level).  ^∗^ *p* < 0.05,  ^∗∗^ *p* < 0.01, and  ^∗∗∗^ *p* < 0.001.

**Figure:**
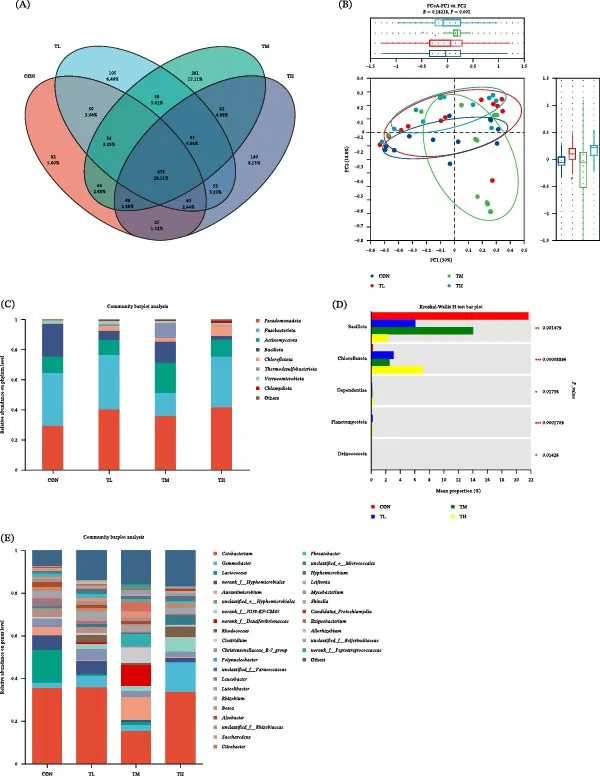

**Figure:**
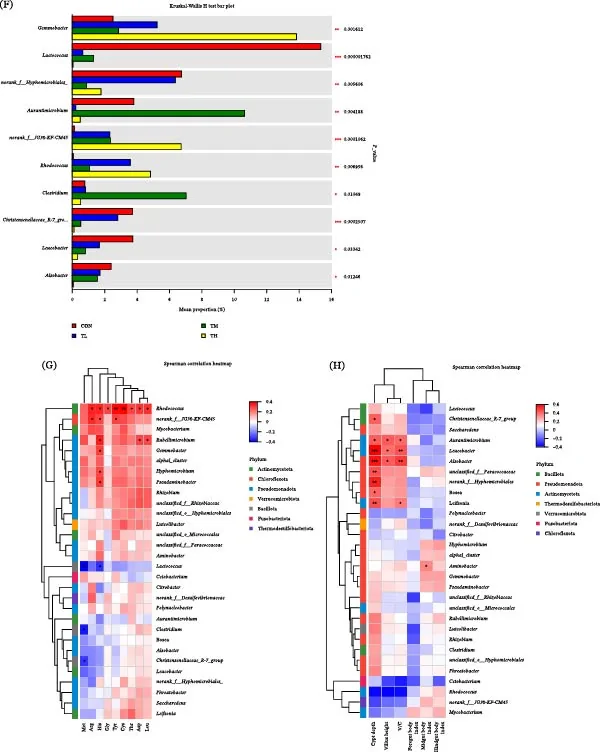

Beta‐diversity, visualized in Figure 6B via PCoA of Bray–Curtis distances, revealed that microbial points from different treatments mapped to discrete, partially overlapping quadrants, implying that SBZ supplementation did not impose a categorical restructuring of community membership or abundance.

At the phylum level (Figure 6C), *Pseudomonadota* and *Fusobacteriota* dominated all groups; Figure 6D highlights significant enrichment of Bacillota, Chloroflexota, Dependiae, and Planctomycetota under SBZ (*p*  < 0.05). Genus‐level profiling (Figure 6E) identified Cetobacterium, Gemmobacter, Lactococcus, Hyphomicrobium, Aurantimicrobium, norank_f_JG30‐KF‐CM45, Desulfovibrionaceae, Rhodococcus, and Clostridium as the principal taxa; among these, Gemmobacter, Lactococcus, Hyphomicrobium, Aurantimicrobium, norank_f__JG30‐KF‐CM45, Rhodococcus, Clostridium, Christensenellaceae, Leucobacter, and Alsobacter exhibited significant differential abundance (*p*  < 0.05, Figure 6F).

Correlation analyses (Figure 6G) linked specific bacteria to muscle amino acid profiles: *Rhodococcus* correlated positively with Arg, His, glycine, threonine, aspartate, and leucine (*p*  < 0.05) and very strongly with tyrosine and cysteine (*p*  < 0.01); *norank_f__JG30-KF-CM45* associated with Arg, His, and tyrosine (*p*  < 0.05); *Rubellimicrobium* with His, aspartate, and leucine (*p*  < 0.05); and *Gemmobacter*, *Hyphomicrobium*, and *Pseudaminobacter* each correlated positively with His (*p*  < 0.05). Conversely, *Lactococcus* displayed a strong negative relationship with methionine (*p*  < 0.01) and a moderate negative link with His (*p*  < 0.05); both *Clostridium* and *Christensenellaceae_R-7_group* were negatively associated with methionine (*p*  < 0.05).

Figure 6H further integrates microbial signatures with intestinal architecture: *Christensenellaceae_R-7_group* correlated positively with VH (*p*  < 0.05); *Aurantimicrobium* with VH, CD, and the villus‐to‐crypt ratio (*p*  < 0.05); *Leucobacter* and *Alsobacter* showed strong positive correlations with VH and the villus‐to‐crypt ratio (*p*  < 0.01) and moderate positive correlations with CD (*p*  < 0.05); and *unclassified_f__Paracoccaceae*, *norank_f__Hyphomicrobiales*, *Bosea*, and *Leifsonia* were positively associated with VH (*p*  < 0.05), while *Leifsonia* additionally correlated with CD (*p*  < 0.05). *Aminobacter* associated positively with mid‐intestinal somatic index (*p*  < 0.05), *Rubellimicrobium* and *Luteolibacter* with VH (*p*  < 0.05), *Cetobacterium* negatively with CD (*p*  < 0.05), and *Rhodococcus* positively with VH and the villus‐to‐crypt ratio (*p*  < 0.05) yet also very strongly with CD (*p*  < 0.01).

## 4. Discussion

The Inner Mongolia Autonomous Region, China’s primary mutton production hub, possesses abundant sheep blood byproduct resources that remain underutilized. This not only constitutes a significant resource waste but also potentially exacerbates ecological pollution. Enzymatically hydrolyzed sheep blood yields nutrient‐dense bioactive compounds that enhance animal growth by modulating key factors in signal transduction pathways to promote cellular synthesis. The core objective of aquaculture is to maximize economic benefits through enhanced growth performance, optimized meat quality, and improved nutritional composition while ensuring fish health. This study demonstrated that supplementation of common carp (*Cyprinus carpio*) basal diets with SBZ significantly increased WGR, daily weight gain, SGR, and CF. These results confirm the zymolysate’s efficacy in promoting growth and enhancing feed utilization efficiency; the improved organ and intestinal indices (consistent with findings in *Atlantic cod* (*Gadus morhua*) *larvae* [15] and *Atlantic salmon* (*Salmo salar*) *larvae* [16]) further indicate that SBZ boosts nutrient absorption by prolonging digesta retention time, thereby elevating overall growth performance and health status. The study demonstrated significantly increased muscle concentrations of both essential and nonessential amino acids (particularly His and Arg) in supplemented groups. Whole‐body composition analysis revealed concurrent optimization of crude protein and crude fat levels, consistent with protein deposition patterns observed in sea bass high‐protein diet trials. This indicates that SBZ enhances protein turnover efficiency, promotes nutrient accumulation, and supports healthy physiological development. Muscle constitutes the primary edible portion of common carp (*C. carpio*). Texture properties are crucial sensory attributes that determine consumer acceptance of food products [17]. These properties primarily include hardness, springiness, cohesiveness, gumminess, chewiness, and shear force. They are closely associated with muscle composition, such as moisture, fat, collagen content, and myofibrillar proteins. In this experiment, the TM group exhibited significantly higher cohesiveness compared to the other three groups, indicating that 0.6% SBZ enhanced the binding force between muscle fibers. The gumminess of the TM group was significantly higher than that of the TL group, and the chewiness of the TM group was significantly greater than that of both the CON and TL groups. These results demonstrate that the 0.6% dose significantly increased the muscle’s resistance to deformation energy. Resilience was also the highest in the TM group, significantly surpassing both the TL and TH groups. The reduced resilience observed at the high dose (TH) may result from excessive small peptides interfering with the myofibrillar network. Collectively, the results indicate that 0.6% SBZ supplementation produced a chewier texture—characterized by synchronous increases in hardness, gumminess, chewiness, and resilience—while also achieving optimal cohesiveness. This outcome aligns with the observed growth performance (where TM showed optimal results), suggesting that the moderate 0.6% SBZ supplementation level not only promotes growth but also improves the textural quality and palatability of the muscle.

Serum biochemical parameters primarily reflect the metabolism of the three major nutrients (proteins, lipids, and carbohydrates) and the functional status of the liver and kidneys [18]. Among these, TP and ALB are critical mediators of protein metabolism. TP indicates the efficiency of protein deposition, whereas ALB—primarily synthesized in the liver—regulates plasma osmotic pressure and facilitates the transport of metabolites. Our results demonstrated increased serum ALB and TP concentrations in common carp fed the SBZ diet. Contrasting with reports by PENG that condensed tannins (≤2 g/kg diet) enhanced glycolipid metabolism while improving hepatic antioxidant and immune responses in sea bass, the present findings suggest a distinct mechanism. This divergence may be attributable to the high iron ion content in SBZ, which potentially augments the utilization efficiency of relevant components, enhances protein metabolic capacity, and contributes to immune system stability.

The intestinal tract, being the primary site of pathogen invasion in fish, critically relies on structural integrity for healthy development [19]. Key morphological indicators of intestinal absorptive capacity include VH, CD, and their ratio (VH/CD). Increased VH correlates with expanded absorptive surface area and enhanced nutrient uptake, while reduced CD indicates accelerated cellular maturation and secretory function. An elevated VH/CD ratio signifies improved digestive/absorptive capacity and mucosal health. The present study demonstrated that dietary supplementation with SBZ significantly increased VH and VH/CD while decreasing CD in common carp. Collectively, these results confirm that the zymolysate enhances intestinal morphological integrity, thereby optimizing nutrient assimilation.

Intestinal barrier function, mediated by the mucosal monolayer, selectively permits nutrient absorption while preventing systemic translocation of pathogens, toxins, and harmful macromolecules [20]. TJPs—including *ZO-1*, *Occludin*, and Claudins—localized at epithelial apical junctions regulate paracellular permeability [21]. They establish selective channels (<200 Da) while restricting pathogen entry, thereby maintaining barrier selectivity, preventing aberrant immune activation, and supporting nutrient/waste exchange [22]. SBZ significantly upregulated intestinal gene expression of *ZO-1*, *Occludin*, and *Claudin-1* in common carp. This TJP enhancement aligns with observations in grass carp (*Ctenopharyngodon idella*) fed probiotics [23] and zebrafish (*Danio rerio*) receiving probiotics [24]. These concordant findings indicate that SBZ reinforces intestinal barrier integrity through TJP modulation.

Amino acids serve as essential nutritional mediators and energy substrates for the intestinal mucosa. Their absorption is governed by epithelial transporters, whose expression dictates systemic amino acid utilization [25]. The present study observed significant upregulation of genes encoding *EAAT*, *CAT*, and *PepT1* in SBZ carp. *PepT1*, widely expressed in intestinal, renal, biliary, and pancreatic tissues, facilitates ditripeptide absorption and transport. These results correspond with increased *LAAT1*, *CAT2*, and *CAT1* in hybrid grouper fed enzymatically digested poultry byproducts [26]. Notably, the SBZ induced significantly stronger *PepT1* activation than poultry byproducts, potentially attributable to its unique bioactive composition. This coordinated upregulation of amino acid and peptide transporters demonstrates the zymolysate’s capacity to enhance nutrient utilization efficiency through trans‐epithelial transport mechanisms.

The gut microbiota serves as a critical indicator of animal health, with its dynamic equilibrium intrinsically linked to microbial composition and distribution [27]. α‐Diversity (Chao1/ACE for richness and Shannon/Simpson for richness and evenness) and β‐diversity (intergroup compositional dissimilarity) are key metrics of gut microbial homeostasis. The present study revealed significantly elevated ACE, Chao1, and Shannon indices alongside reduced Simpson index in the SBZ group relative to CONs, with no significant β‐diversity differences observed. These findings align with Yang et al. [28] where enzymatically hydrolyzed poultry protein similarly enhanced α‐diversity metrics without altering community structure. Collectively, these results demonstrate that SBZ supplementation enhances intestinal microbial diversity and species richness in common carp (*C. carpio*) while preserving fundamental community composition and maintaining enteric microbial homeostasis.

In common carp (*C. carpio*), gut microbiota is dominated by Proteobacteria (metabolically versatile) and Fusobacteria (amino acid‐fermenting anaerobes mediating host‐microbe interactions); SBZ supplementation reshaped this community toward a healthier profile. At the genus level, core taxa such as *Cetobacterium*, *Gemmatimonas*, and *Lactococcus* are widely recognized in fish intestines [29], with genera including *Bifidobacterium*, *Bacillus*, *Lactococcus*, and *Lactobacillus* acknowledged as probiotics conferring intestinal health benefits [30, 31]. The present study demonstrates that dietary SBZ improves intestinal health in carp by suppressing pathogenic bacteria proliferation and maintaining microbial homeostasis—consistent with Echave et al.’s [32] findings that protein zymolysates enhance beneficial microbiota abundance and barrier function.

Analysis of Top30 genera via correlation heatmaps with muscle amino acids and intestinal indices elucidates potential mechanisms through which the zymolysate modulates amino acid metabolism and gut health. *Rhodococcus*, an intestinal beneficial genus, facilitates nutrient assimilation and inhibits pathogens. Our results reveal a positive correlation with valine, leucine, and isoleucine metabolism, suggesting enhanced enterocyte energy metabolism. *Rubellimicrobium* regulates amino acid equilibrium through essential amino acid secretion, branched‐chain fatty acid synthesis, and probiotic proliferation. These findings, corroborating Naya‐Català et al. [33], confirm the functional prominence of these genera under dietary conditions, ultimately elevating amino acid transport efficiency and promoting protein synthesis and somatic growth. *Christensenellaceae_R-7_group*—a Firmicutes‐affiliated dominant genus—exhibits abundance variations closely linked to intestinal structural and functional parameters. Studies by Chen et al. [34] establish that its increased abundance positively correlates with elevated VH and reduced CD, consistent with our observations. This evidence collectively indicates that SBZ stimulates carp enterocyte proliferation, improves histological architecture, mitigates intestinal inflammation, and sustains gastrointestinal health.

## 5. Conclusions

Under the experimental conditions, dietary supplementation with SBZ in common carp (*C. carpio*) basal diets enhanced growth performance, augmented intestinal nutrient absorption, and stabilized the enteric microenvironment—collectively optimizing intestinal health status. The 0.6% inclusion level yielded optimal integrated effects, achieving concurrent improvements in growth performance, health enhancement, and efficient resource utilization. These results highlight the potential of this technology to transform sheep blood waste into valuable aquafeed ingredients.

## Author Contributions

**Jingjing Bao**: writing – original draft, formal analysis, data curation. **Yujun Jiang**: writing – review and editing, formal analysis. **Xusheng Hao**: methodology, investigation. **Xuelian Feng**: resources, methodology. **Mengyuan Zhang**: resources, methodology. **Yina Qiao**: resources, methodology. **Xin Hou and Xiaoyi Xu**: methodology. **Yilin Yang**: methodology, investigation. **Yingchun Liu**: supervision, methodology, conceptualization. **Yuhong Zhang**: supervision, methodology, conceptualization. **Feng Gao**: writing – review and editing, project administration, funding acquisition.

## Funding

This research was supported by the Interdisciplinary Research Fund of Inner Mongolia Agricultural University (Grant BR231402), the Inner Mongolia Autonomous Region Science and Technology Plan Project (Grant 2023YFHH0030), and the China Agriculture Research System of MOF and MARA (Grant CARS‐38).

## Conflicts of Interest

The authors declare no conflicts of interest.

## Data Availability

The datasets generated and/or analyzed during the current study are available from the corresponding author upon reasonable request.
